# A novel *POLR3A* genotype leads to leukodystrophy type-7 in two siblings with unusually late age of onset

**DOI:** 10.1186/s12883-020-01835-9

**Published:** 2020-06-29

**Authors:** Rosa Campopiano, Rosangela Ferese, Stefania Zampatti, Emiliano Giardina, Francesca Biagioni, Claudio Colonnese, Diego Centonze, Marianna Storto, Fabio Buttari, Edoardo Fraviga, Vania Broccoli, Mirco Fanelli, Francesco Fornai, Stefano Gambardella

**Affiliations:** 1 I.R.C.C.S. I.N.M. Neuromed, via Atinense 18, 86077 Pozzilli, Italy; 2 Molecular Genetics Laboratory UILDM, Santa Lucia Foundation IRCCS, Rome, Italy; 3 Department of Biomedicine and Prevention, University of Rome “Tor Vergata”, Rome, Italy; 4 Dipartimento di Medicina dei Sistemi, Università di Roma Tor Vergata, Rome, Italy; 5 Stem Cell and Neurogenesis Unit, Division of Neuroscience, San Raffaele Scientific Institute, Milan, Italy; 6 CNR Institute of Neuroscience, Milan, Italy; 7 Department of Biomolecular Sciences, University of Urbino “Carlo Bo”, Urbino, Italy; 8 Department of Translational Research and New Technologies in Medicine and Surgery, University of Pisa, Pisa, Italy

**Keywords:** *POLR3A* mutations, Hypomyelinating leukodystrophies, Age of onset, Brain

## Abstract

**Background:**

Leukodystrophies are familial heterogeneous disorders primarily affecting the white matter, which are defined as hypomyelinating or demyelinating based on disease severity as assessed at MRI. Recently, a group of clinically overlapping hypomyelinating leukodystrophies (HL) has been associated with mutations in RNA polymerase III enzymes (Pol III) subunits.

**Case presentation:**

In this manuscript, we describe two Italian siblings carrying a novel *POLR3A* genotype. MRI imaging, genetic analysis, and clinical data led to diagnosing HL type 7. The female sibling, at the age of 34, is tetra-paretic and suffers from severe cognitive regression. She had a disease onset at the age of 19, characterized by slow and progressive cognitive impairment associated with gait disturbances and amenorrhea. The male sibling was diagnosed during an MRI carried out for cephalalgia at the age of 41. After 5 years, he developed mild cognitive impairment, dystonia with 4-limb hypotonia, and moderate dysmetria with balance and gait impairment.

**Conclusions:**

The present study provides the first evidence of unusually late age of onset in HL, describing two siblings with a novel *POLR3A* genotype which showed the first symptoms at the age of 41 and 19, respectively. This provides a powerful insight into clinical heterogeneity and genotype-phenotype correlation in *POLR3A* related HL.

## Background

Leukodystrophies are heterogeneous familial disorders primarily affecting the white matter, which are associated with cytological abnormalities in glial cells and myelin sheath. They are classified as hypomyelinating or demyelinating depending on disease severity at MRI. Many leukodystrophies have been now genetically well characterized [1].

Among leukodystrophies, a group of clinically overlapping hypomyelinating leukodystrophies (HL) has been recently associated with mutations in polymerase III enzyme (Pol III) subunits [2–5]. Mutations in Pol III subunits *POLR3A* and *POLR3B* have been related to HL. Since early identification of mutations in *POLR3A*, more than 100 mutations in *POLR3A*, *POLR3B,* and *POLR1C* have been identified in over 130 patients with *POLR3*-HL [6]. These variants may occur at any given coding exon or splicing junctions within these genes, which indicates a high degree of mutations heterogeneity [7].

In these patients, MRI features an abnormally low amount of myelination, which is defined as hypomyelination [8]. The most common Pol III leukodystrophies are related to a clinical syndrome named 4H, meaning that leukodystrophies occur as hypomyelination along with hypogonadotropic hypogonadism and hypodontia. Neurological features include neurodevelopmental delay and motor symptoms which witness for cerebellar, pyramidal, and basal ganglia alterations such as ataxia, nystagmus, paresis, dystonia, respectively. At the onset, motor symptoms may not be present and the disease appears as cognitive impairment. The disease extends to non-neurological features including dental, endocrine, and visual abnormalities [2, 9–11].

4H leukodystrophy being described so far leading to an onset before the age of 5 with a rapid and severe clinical course, and severe motor and intellectual impairment related to a limited life expectancy [10].

The present study provides the first evidence of unusually late onset form of HL, describing two siblings with a novel *POLR3A* genotype which showed the first symptoms at the age of 41 and 19, respectively.

## Case presentation

### Genetic analysis

Genomic DNA was isolated from peripheral blood leukocytes according to standard procedures. Clinical exome sequencing considering 4800 human genes (TruSight One Sequencing Panels, Illumina) was performed on MiSeq platform (Illumina). Variant Studio was used for annotation and characterization of variants. Manual examination and visualization of the sequence data were performed by the Integrative Genomics Viewer v.2.3, while the selection of potentially pathogenic variants by Tgex software (LifeMap Sciences). Mutations were re-sequenced by Sanger sequencing (ABI 3130xl Genetic Analyzer, Applied Biosystems). Variants were tested by using public databases like NHLBI Exome Sequencing Project (ESP) (http://evs.gs.washington.edu/EVS/) and ExAc (http://exac.broadinstitute.org/) and called according to HGVS nomenclature. The new variant NM_007055:c.[2325C > G]; NP_008986:p.(Asn775Lys) was recorded in ClinVar (http://www.ncbi.nlm.nih.gov/clinvar) with ID: SUB6063757.

### Genetic case

We report two Italian siblings with a clinical and instrumental phenotype indicating a diagnosis of HL.

**Fig. 1:**
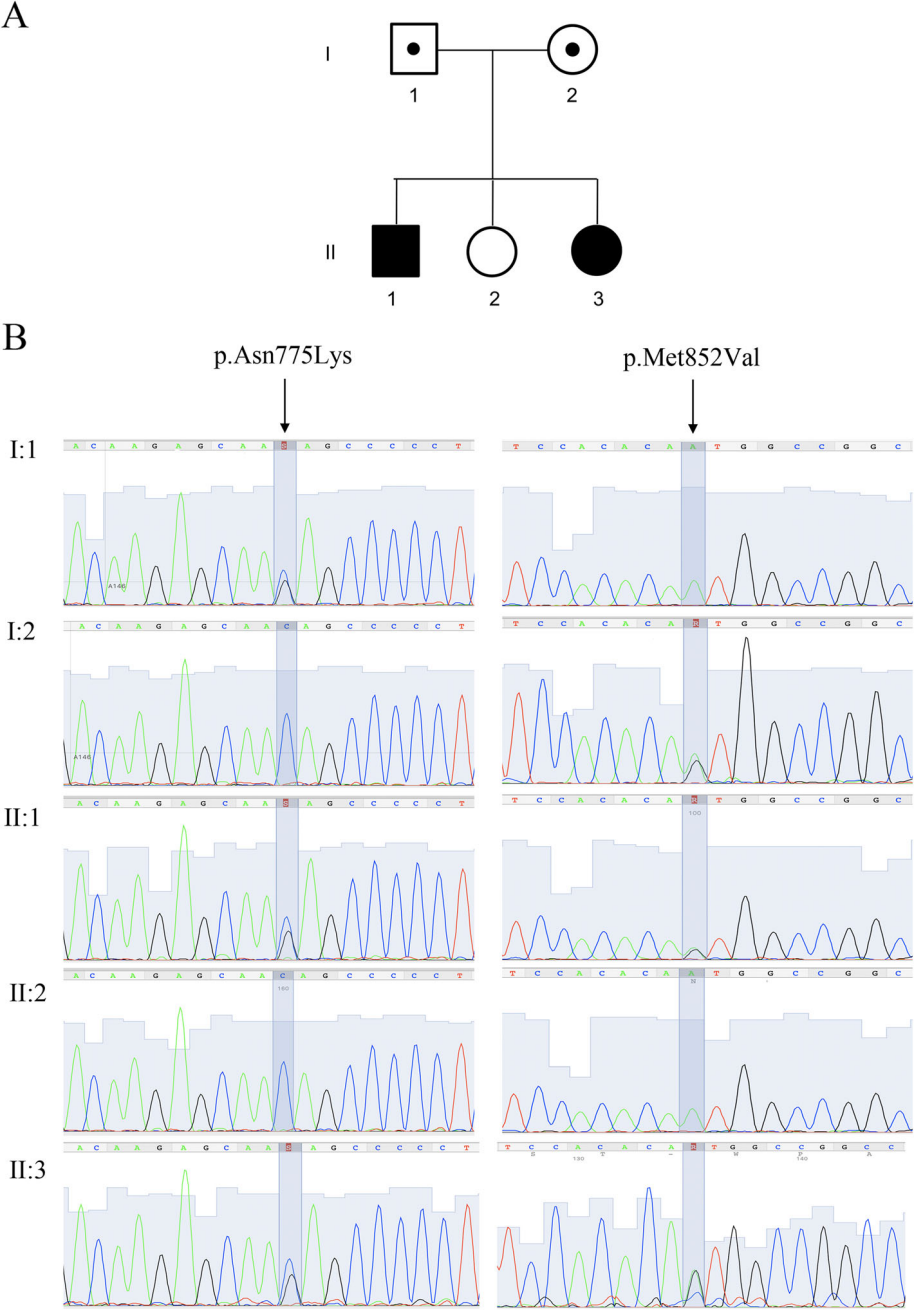
Genetic analysis. **a** Pedigrees of the investigated proband siblings. (I:1) and (I:2) are asymptomatic carrier parent. Probands (II:1) and (II:3), are affected siblings; (II:2) is healthy sister. **b** Co-segregation analysis of p.Asn775Lys (NP_008986), c.2325C > G (NM_007055) in exon 17 and p.Met852Val (NP_008986), c.2554A > G (NM_007055) (rs267608671) in exon 19 of gene *POLR3A* (OMIM #614258). Sequence analysis is shown for father (I:1) is heterozygous carrier of p.Asn775Val variant and wild type for p.Met852Val variant, mother (I:2) is wild type for p.Asn775Val variant and heterozygous carrier of p.Met852Val variant, probands (II:1) and (II:3) are heterozygous for both variants, helthy sister is wild type for both variants

**Fig. 2:**
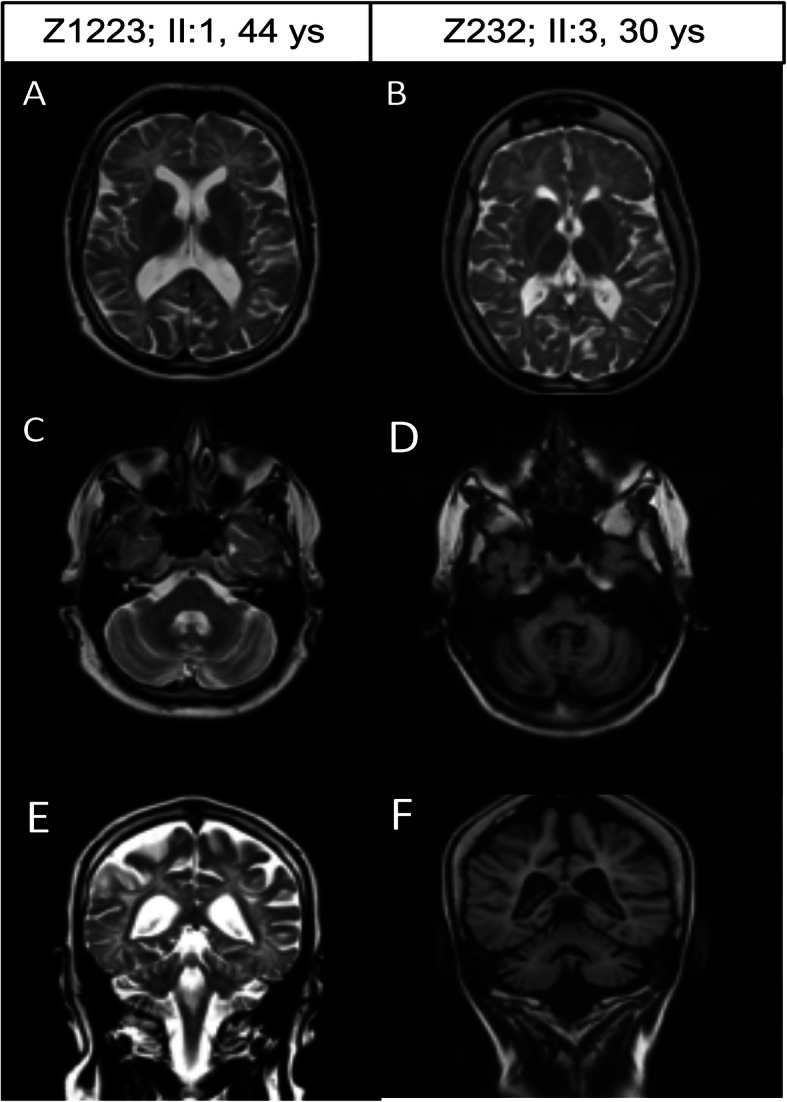
MRI of proband siblings. Axial and Coronal T2 are shown for male proband, Z1223 (on the left, images **a**, **c**, **e**) at the age of 44 years old, and the female proband, Z232 (on the right, images **b**, **d**, **f**) at the age of 32 years old. Axial T2 weighted MRI of brain at the level of basal ganglia (A and B: Turbo Spin Echo). Images show in male proband an evident dilatation of the supratentorial ventricular system, diffused hyperintensity of the white matter mainly in the frontal site (**a**). The female proband possesses the same pattern, but diffused hyperintensity of the white matter is present in both frontal and posterior lobe. A marked hyperostosis is evident (**b**). Axial T2 weighted MRI of brain at the level of posterior cranial fossa (C and D: T2 FLAIR). Images show in male proband an hyperintensity of the middle cerebellar peduncle and dilatation of the fourth ventricle of the prepontine cistern and subarachnoid spaces (**c**). The female proband possesses the same pattern although each alteration is more severe (atrophy of cerebellar peduncles, enlargement of the fourth ventricle pre-pontine cistern and subarachnoid spaces) (**d**). Coronal T2 weighted (E = T2 Turbo Spin Echo; F = T1 Spin Echo). Both male (**e**) and female (**f**) proband show a marked enlargement of cortical subarachnoid spaces, enlargement of middle cell of the lateral ventricle enlargement of the fourth ventricle and widespread hyperintensity of the white matter

The female proband (Z232; II:3) (Fig. 1a) had bilateral dysplasia of the hip at birth, for which she underwent correction surgery. She did not carry any dental abnormalities. Before the age of 19, she had primary amenorrhea which was treated by hormone replacement therapy. No gynecological and endocrinological evaluation was available at the time of our first evaluation. At this time a delayed psychomotor development was detected. The neurological exam reported progressive gait alterations, while brain MRI was compatible with leukodystrophy (Fig. 2).

In detail, atrophy in the hemispheric white matter involving the capsules was detected. When considering the age, cortical atrophy was pronounced. A follow-up MRI at the age of 26, reported skull hyperostosis and widespread hypomyelination in the context of both hemispheres and cerebellar atrophy including cerebellar peduncles. These findings were similar to those reported by Minnerop et al. and Rydning et al. [12, 13]. In the same year, EEG activity reported slight abnormalities consisting of reduced theta waves voltage. The motor impairment progressed gradually leading to hospitalization at the age of 27 and again at the age of 29. During the first hospitalization, she had a severe spastic and ataxic para-paretic gait. She also had weakness and dysarthria along with cerebellar symptoms such as nystagmus. The patient did not show hypodontia, delayed dentition, or other dental abnormalities.

MRI carried out at the age of 29 and 32 showed progressive thinning of the corpus callosum and confirmed the cortical/subcortical atrophy which included the brainstem and cerebellum including cerebellar peduncles. Motor impairment progressed and she was obliged in a wheelchair. Nystagmus was evident in the horizontal gaze, while conjugated eye movement was slowed both in the horizontal and vertical planes. Ataxia was manifest with dysmetria adiadocokinesia, spasticity was concomitant with a loss of strength and pathological stretch reflex at four limbs. Other neurological signs included left foot myoclonus and reduced pallesthesia. No peripheral nerve alteration was detected at ENG. At the age of 32 facial and buccal apraxia were manifest, and MRI became difficult for movement artifact but a marked and atrophy of the white matter (worsened compared to the previous exam) was detected both in the white matter (including the corpus callosum) and cortical gyri cerebellum and brainstem along with connecting peduncles. No contrast enhancement was documented. (Fig. 2, images b, d, f). Due to the severe motor worsening, the patient was unable to repeat the endocrinological evaluation at our Institute.

The male proband (Z1223; II:1) (Fig. 1a), did not report any specific symptom in childhood. At the age of 38, he had a headache episode with a loss of consciousness, which required an MRI. At that time only MRI was abnormal and it revealed a moderate increase of the ventriculo-cisternal system and white matter hyperintensity which were associated with a slight, though non-specific EEG alterations. At that time, no other clinical feature was reported. After a second severe headache episode, at the age of 41, the patient was hospitalized and a control MRI revealed a progression in white and grey matter atrophy. Resonance spectroscopy showed a pattern that was compatible with HL consisting of slight NAA reduction in the absence of any other alteration. The patient had normal score at Mini Mental State Examination (MMSE 28/30), however neuropsychological evaluation showed a slight impairment memory functions The neurological exam evidenced increased muscle tendon reflexes including the masseter. Despite a spastic muscle tone, muscle strength was not reduced. No cerebellar or sensory symptoms were evident at that time. At the age of 44, the patient’s MRI was typical for HL as shown by the loss of white matter homogeneity in the forebrain (Fig. 2, images a, c, e). At present, at the age of 46, the patient developed dystonia in the context of 4-limb hypotonia with moderate dysmetria and difficulty in balance and gait.

To support clinical and instrumental data suggestive for HL, genetic analysis was carried out by clinical exome sequencing (4800 genes related to genetic disorders) in both siblings. In detail, sequencing was performed in the female proband (Z232; II:3) at the age of 30, in the presence of clinical and instrumental evidence. Data analysis identified compound heterozygous mutations NM_007055:c.[2325C > G]; NP_008986:p.(Asn775Lys) and NM_007055:c.[2554A > G]; NP_008986:p.(Met852Val) (Fig. 1b), which were consistent with autosomal recessive inheritance. Variants were validated using Sanger sequencing**,** and tested in the male proband (Z1232; II:1) for purposes of segregation analysis, at the age of 44. At that time, MRI was carried out for other purposes (headache), and slight clinical symptoms were not recognized but they were re-considered in a retrospective anamnesis.

Segregation analysis confirmed that the mutations were inherited by heterozygous carrier parents. p.Asn775Lys variant (Fig. 1b), present in the father (I:1), were not observed in public or internal variant databases. p.Met852Val (Fig. 1b), present in the mother (I:2), was already reported in HL [9].

## Discussion and conclusions

Clinical features of HL patients with mutations in *POLR3A* or *POLR3B* consist of neurological, dental, ophthalmological, and endocrine alterations. In each patient at least one alteration is present.

Neurological signs and symptoms include motor and cognitive delay or impairment, cerebellar symptoms (mostly ataxia and nystagmus). Cerebellar atrophy is reported in more than 80% of patients, while only a few patients have extrapyramidal signs (mainly dystonia, and tremor). Pyramidal symptoms are common (paresis, abnormal stretch reflex; spastic tone), and 20% of patients develop epileptic seizures. There is a constantly cognitive impairment [10, 14–16]. All patients, after some years, do not achieve independent walking and use a wheelchair.

Dental abnormalities, such as delayed dentition and hypodontia are common (more than 80%). Most patients present endocrine (hypogonadotropic hypogonadism) and/or ophthalmological abnormalities (optic atrophy and myopia).

In this manuscript, we describe two Italian siblings with HL type 7 supported by clinical, instrumental, and genetic analysis. Patients bearing this novel genotype in the *POLR3A* gene become symptomatic only in late adolescence or early adulthood [10] considering both clinical symptoms and MRI pattern.

The typical MRI pattern for *POLR3*-related leukodystrophy is characterized by diffuse hypomyelination associated with relative T2 hypointensity of the ventrolateral thalamus, globus pallidus, optic radiation, corticospinal tract at the level of the internal capsule and dentate nucleus, cerebellar atrophy, and thinning of the corpus callosum [10, 14, 17, 18]. Differences have been reported comparing *POLR3A* and *POLR3B* patients [14, 16]. Patients with *POLR3B* mutations show small cerebellum (hemispheres and vermis) with thin folia and enlarged fissures, with cerebellar atrophy suggested by the decreased cortical thickness and diminished underlying white matter. MRI in patients with *POLR3A* mutations revealed significantly slighter changes in the cerebellar hemispheres and vermis with only a slight hypomyelination in the centrum semiovale and cerebellar white matter [19]. However, *POL3A* may lead to cerebellar atrophy which extends at least to the brainstem superior cerebellar peduncles [12].

In this regard, this genotype may resemble those types of *POL3A* leukodystrophy which features evident sub-tentorial atrophy. In line with this, a diffuse brainstem involvement was recently described [17].

The first variant identified, p.Met852Val could reduce RPC1-RPC2 protein interaction, thus impairing RNA Pol III proper function. This variant has already been reported in HL patients. In detail, it was reported once in association with p.Arg873AlafsX878 in two 4H female (onset at the age of 12), and with p.Arg140X in a female affected by leukodystrophy with oligodontia (onset at the age of 3) [9].

The second variant, p.Asn775Lys could reduce RPC1 flexibility impairing RNA Pol III proper function. This variant has never been identified in HL, and is not reported in the public databases. Interestingly, Bernard et al. described the mutation p.Asn775Ile [9]. This variant affects the same amino acid but causes an Asparagine to Isoleucine transition. This variant has been identified in association with p.Asp372Asn in a 4H female patient. This patient has a disease onset a few months after birth, with developmental delay and cognitive regression, dysphagia, hypersalivation, tremor, and cerebellar signs.

A few data are available concerning HL patients with the same genotype. A deep focus on these patients allows speculating that patients carrying the same genotype manifest similar clinical features. For example, in French Canadian patients homozygote for p.Gly672Glu in *POLR3A*, age of onset was usually in infancy or early childhood showing upper motor neuron signs, tremor, cerebellar signs, and cognitive regression. Besides, genotype p.Met852Val / p.Arg873AlafsX878 present age of onset at 12, with cerebellar signs and cognitive regression without developmental delay in two 4H females.

However, the analysis of the family reported in this manuscript demonstrate that, apart from a few rare exceptions, it is not possible to perform genotype-phenotype correlation for variants in *POLR3A* and *POLR3B.*

In conclusion, the genotype reported in the siblings from this manuscript leads to a unique phenotype which is characterized by a delayed disease onset and slow progression (albeit differing of about 20 years). This provides an opportunity to follow up disease progression in such a slowly developing disorder which may allow us to better characterize the neurobiology of disease and improve the quality of life starting from this very same patient.

## Data Availability

Data sharing is not applicable to this article as no datasets were generated or analyzed during the current study.

## Abbreviations

HL: Hypomyelinating leukodystrophies
Pol III: RNA polymerase III enzyme
MMSE: Mild cognitive impairment
